# Supplementary material for: The association of UBAP2L and G3BP1 mediated by small nucleolar RNA is essential for stress granule formation
Source: Commun Biol. 2023 Apr 14;6:415. doi: 10.1038/s42003-023-04754-w (PMC10104854; doi:10.1038/s42003-023-04754-w)

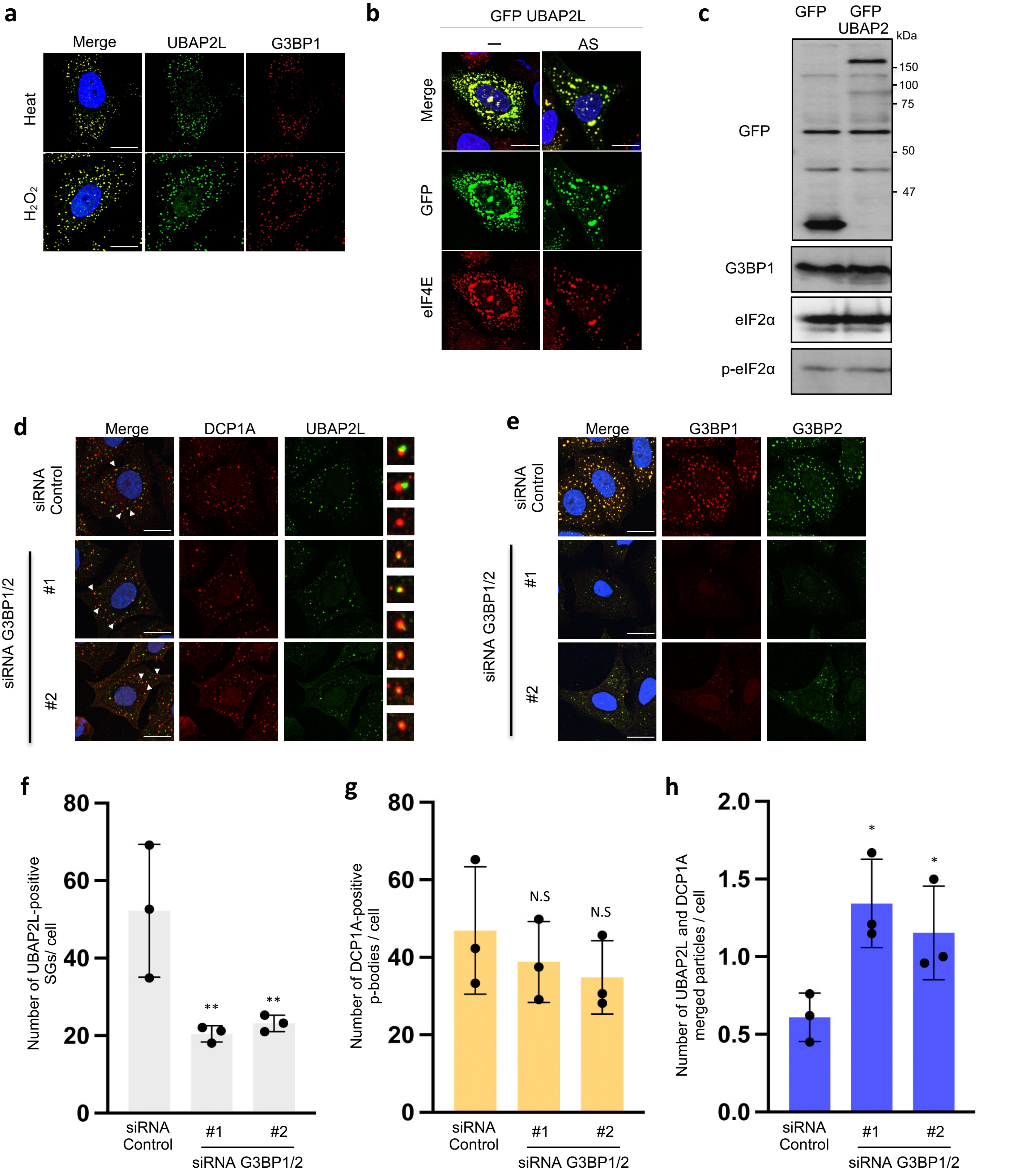

Supplementary Figure 1. UBAP2L is localized to SGs in response to various stress conditions. Related to Figure 1. **a** HeLa cells were treated with heat shock (44°C) for 30 min or 1 mM hydrogen peroxide (H_2O_2) for 1.5 h. The cells were immunostained for UBAP2L and G3BP1 (scale bar=10μm). **b** HeLa cells were transfected with plasmids encoding GFP or GFP-UBAP2L, and 24 h later, the cells were immunostained for GFP and eIF4E with or without 0.5 mM arsenite (AS) treatment for 30 min (scale bar=10μm). **c** Cells were transfected with plasmids encoding indicated cDNA and 24 h later, the cells were lysed and immunoblotted. **d-h** HeLa cells were transfected with control or G3BP1/2 siRNAs. After 72h, the cells were treated with 0.5mM Arsenate for 30min and immunostained with anti-UBAP2L and anti-DCP1A (**d**) or anti-G3BP1 and anti-G3BP2 (**e**) (scale bar=10μm). The numbers of SGs positive for UBAP2L per cell were presented in graph (f). The numbers of PBs positive for DCP1A per cell were presented in graph (**g**). The numbers of merged UBAP2L and DCP1A particles per cell were presented in graph (**h**). Three independent experiments were performed and about 20 cells were evaluated for each experiment ($n = 3$, * $P < 0.05$, N.S. (not significant) $P > 0.05$).

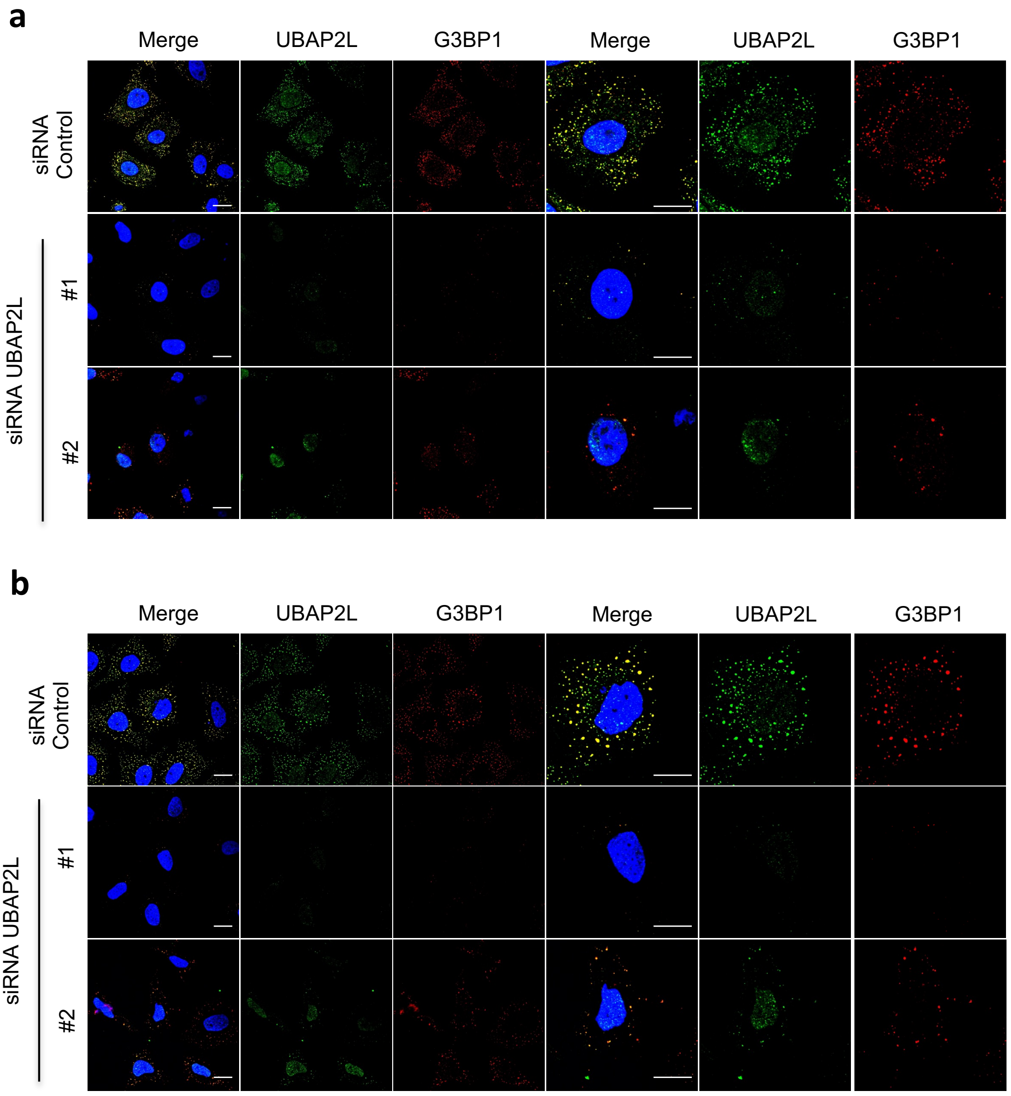

Supplementary Figure 2. UBAP2L depletion suppressed SG assembly by heat and sorbitol treatments.
a.b HeLa cells were transfected with control or UBAP2L siRNAs. After 72h, the cells were treated with heat shock (44℃) for 30min (**a**) or 0.3 M sorbitol for 30min (**b**) and immunostained with anti-UBAP2L and anti-G3BP1 antibodies (scale bar=10μm).

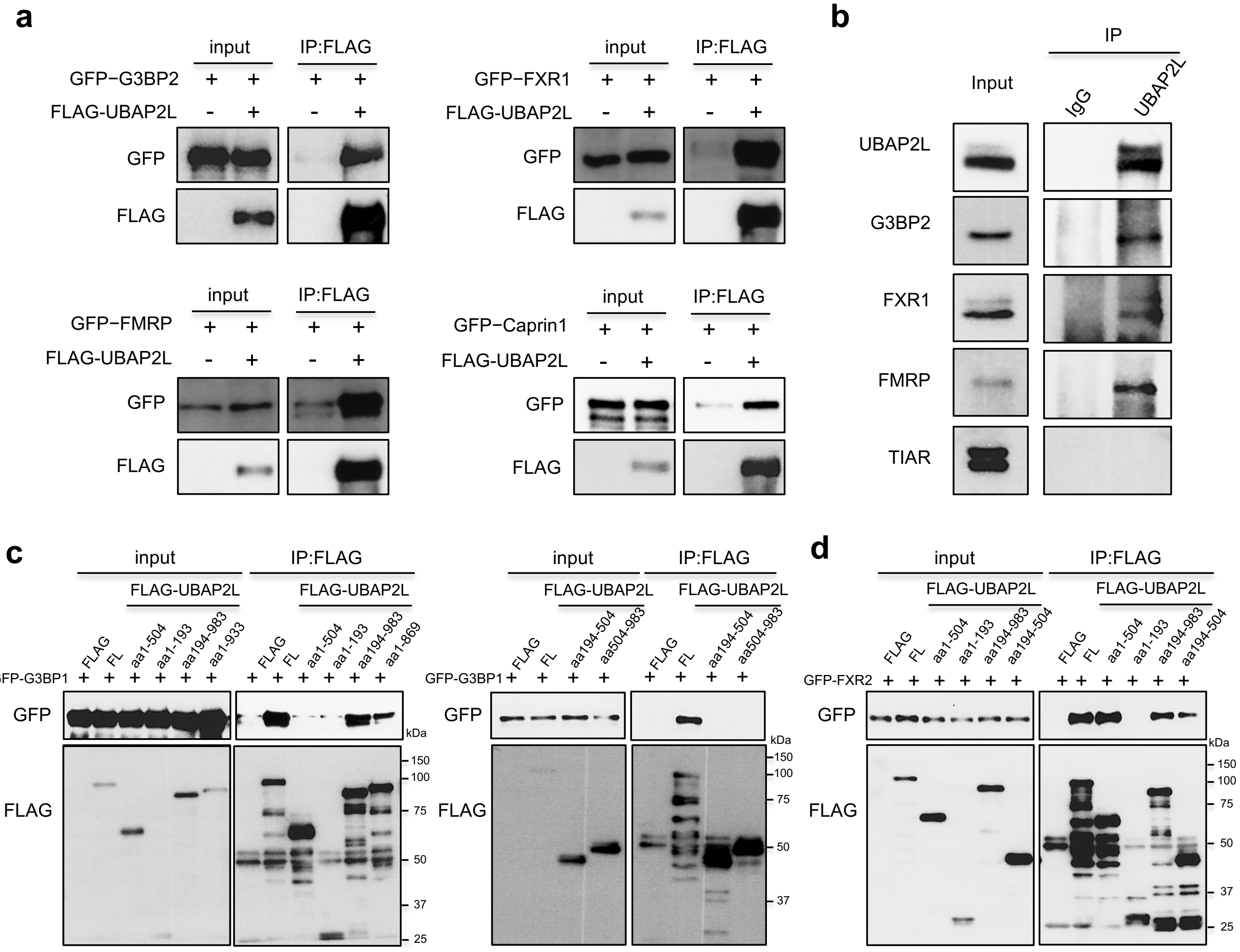

Supplementary Figure 3. UBAP2L interacted with multiple SG localizing proteins.

a 293T cells were transfected with FLAG-UBAP2L together GFP-G3BP2, GFP-FXR1, GFP-FMRP or GFP-Caprin1, and 24 h later, the cells were lysed and immunoprecipitated with an anti-FLAG antibody. The immunoprecipitates were immunoblotted by anti-GFP and anti-FLAG antibodies. **b** HeLa cells were lysed and immunoprecipitated with control or anti-UBAP2L antibodies. The immunoprecipitates were subjected to immunoblot analysis. **c** 293T cells were transfected with GFP-G3BP1 together with FLAG-UBAP2L deletion mutants. After 24 h, cells were lysed and immunoprecipitated with an anti-FLAG antibody and subjected to immunoblotting to probe for GFP or FLAG. **d** 293T cells were transfected with GFP-FXR2 together with GFP-UBAP2L deletion mutants. After 24 h, cells were lysed and immunoprecipitated with an anti-FLAG antibody and subjected to immunoblotting to probe for GFP and FLAG.

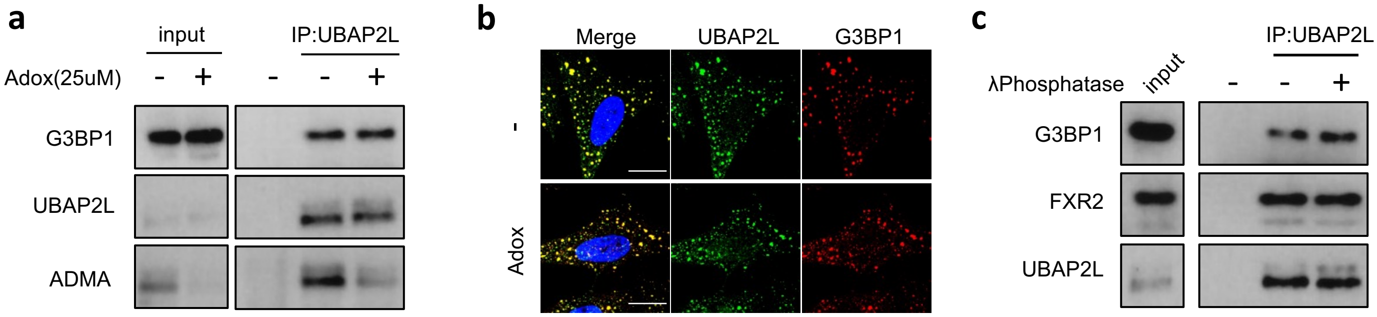

Supplementary Figure 4. Adox and phosphatase treatments did not affect the interaction between UBAP2L and G3BP1. **a** HeLa cells were cultured in the presence or absence of 25uM of Adox for 24 h, and lysed, and immunoprecipitated with an anti-UBAP2L antibody. The immunoprecipitates were immunoblotted with indicated antibodies. **b** Cells were cultured with or without Adox for 24 h, and then treated with 0.5 mM arsenite for 30 min. The cells were immunostained for G3BP1 and UBAP2L (scale bar=10μm). **c** HeLa cells were immunoprecipitated with an anti-UBAP2L antibody, the immunoprecipitates were treated with lambda phosphatase and immunoblotted.

a

RPM(G3BP1)	Accession	Description	Gene Type
61985.91399	RNU2−2	RNA, U2 small nuclear 2	snRNA
60072.56429	SNORD44	small nucleolar RNA, C/D box 44	snoRNA
59113.22164	SNORD49A	small nucleolar RNA, C/D box 49A	snoRNA
57704.62064	SCARNA20	small Cajal body−specific RNA 20	snoRNA
54777.50507	RNU12	RNA, U12 small nuclear	snRNA
50410.84196	SNORD74	small nucleolar RNA, C/D box 74	snoRNA
45382.56323	SNORD3A	small nucleolar RNA, C/D box 3A	snoRNA
44895.95561	RNU5B−1	RNA, U5B small nuclear 1	snRNA
32952.7265	SNORD3C	small nucleolar RNA, C/D box 3C	snoRNA
32092.62619	RNU5A−1	RNA, U5A small nuclear 1	snRNA
⋮	⋮	⋮	⋮
0	SNORD56	Small nucleolar RNA, C/D box 56	snoRNA

b

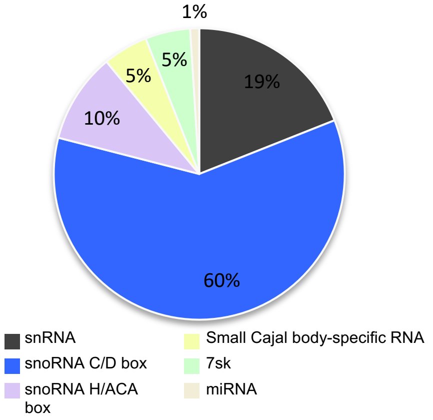

c

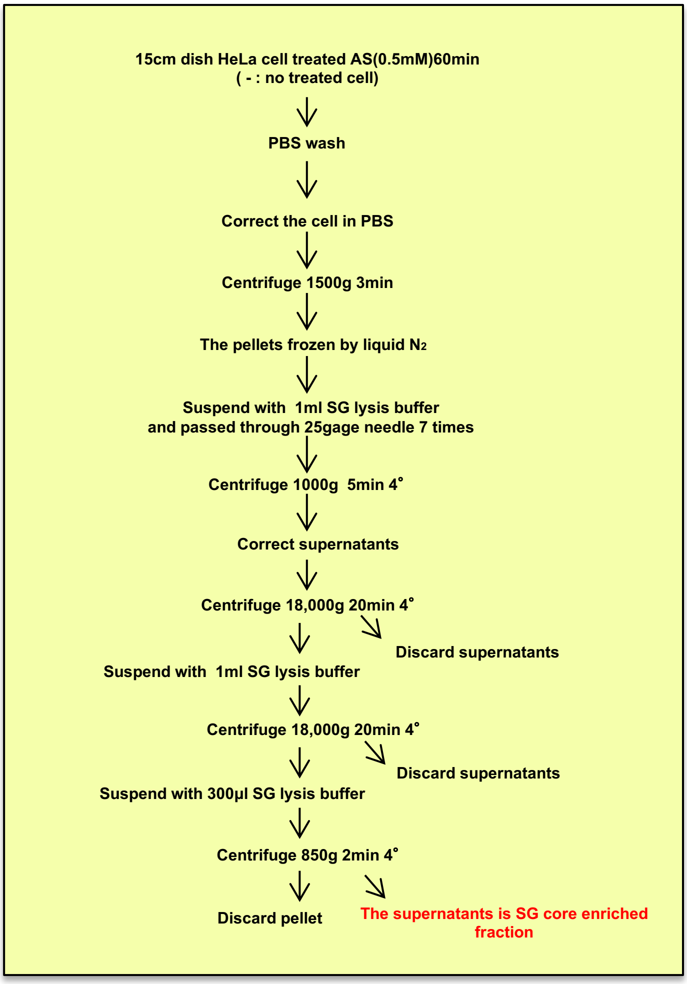

d

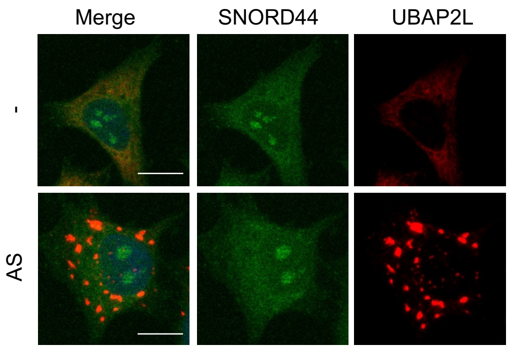

Supplementary Figure 5. The results of small RNA sequencing of G3BP1 RIP.
a The RNAs with the top 10 G3BP1 RPM scores are presented. **b** The percentages of each type of gene of the RNAs with top 100 G3BP1 RPM scores are shown in the circle graph. **c** HeLa cells were treated with or without 0.5 mM arsenite for 30 min. **c** Method for purification of SG core fraction. **d** Cells were fixed and hybridized SNORD 44-DIG probe for overnight, and then cells were immunostained for DIG and UBAP2L.

Supplementary Figure 6. Uncropped blots for each indicating figure.

Uncropped blots for Figure 2b

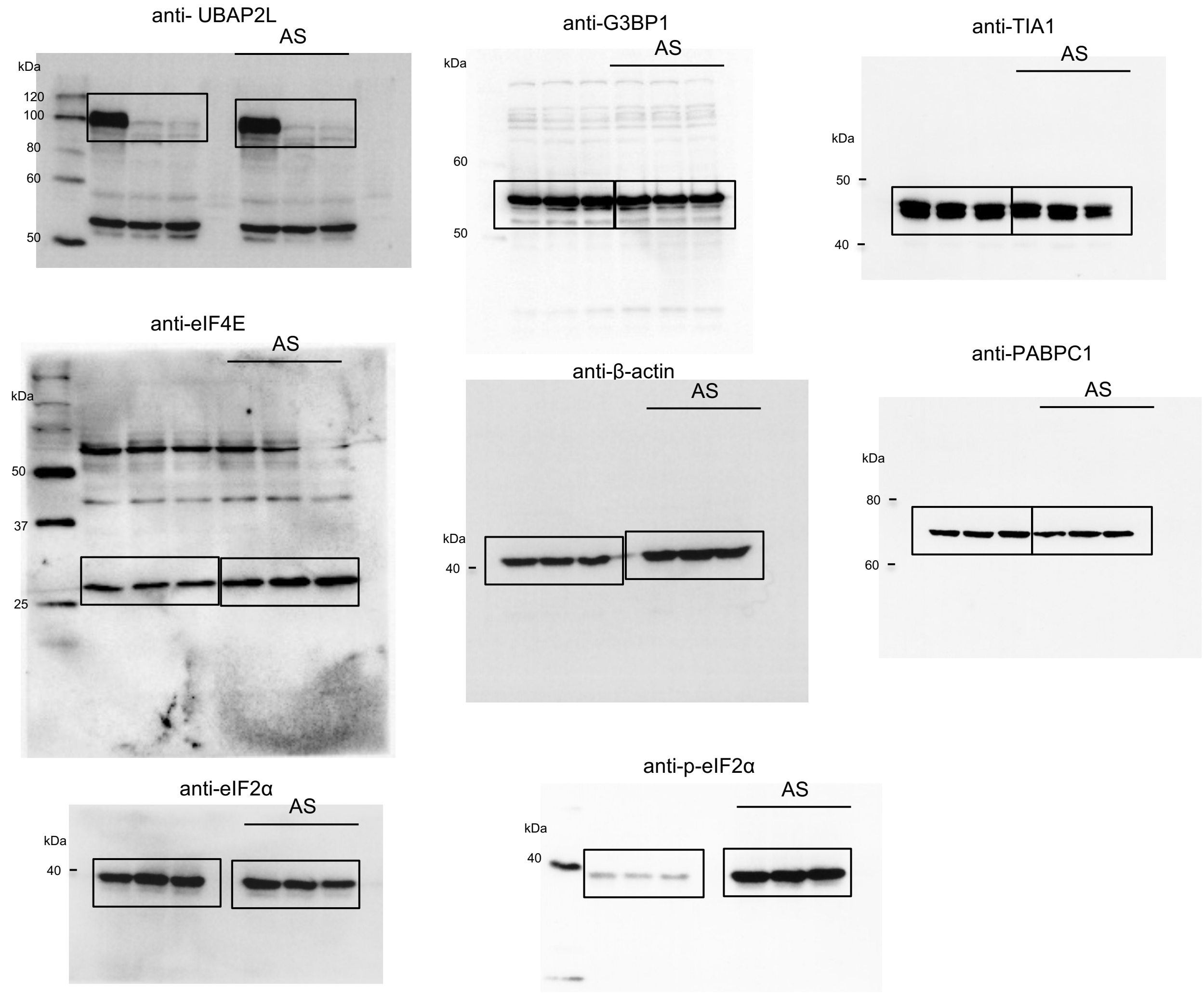

Uncropped blots for Figure 2c

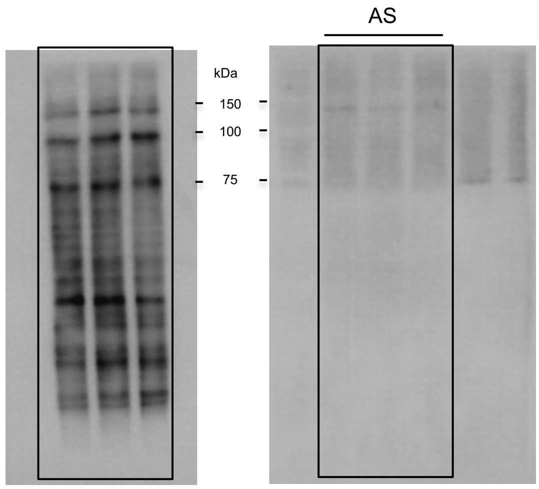

Uncropped blots for supplementary Figure 1C

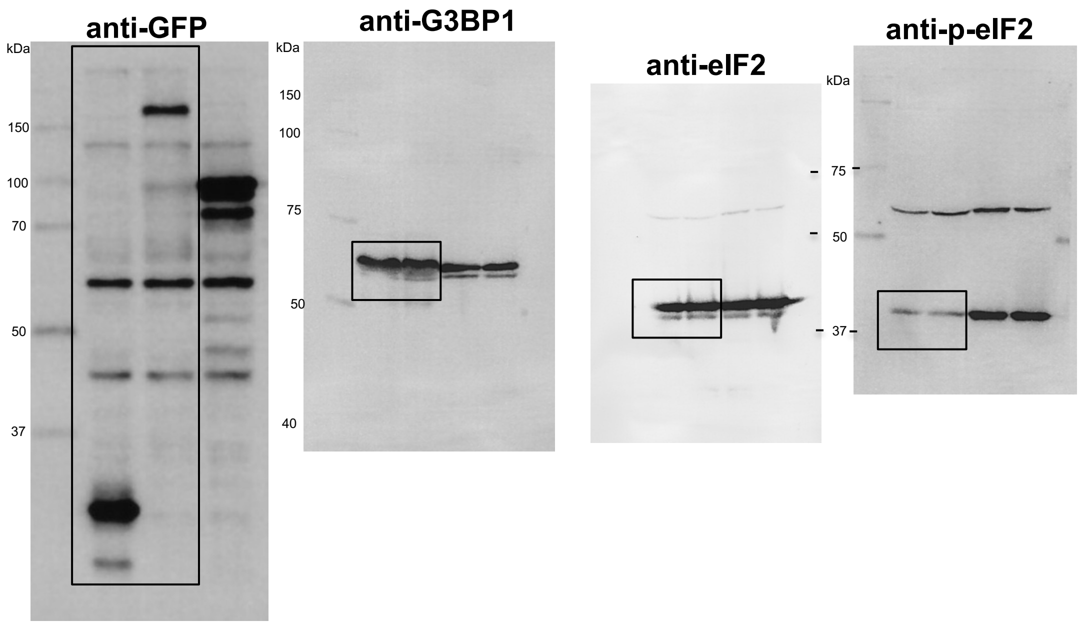

Uncropped blots for Figure 3b and supplementary Figure 3a

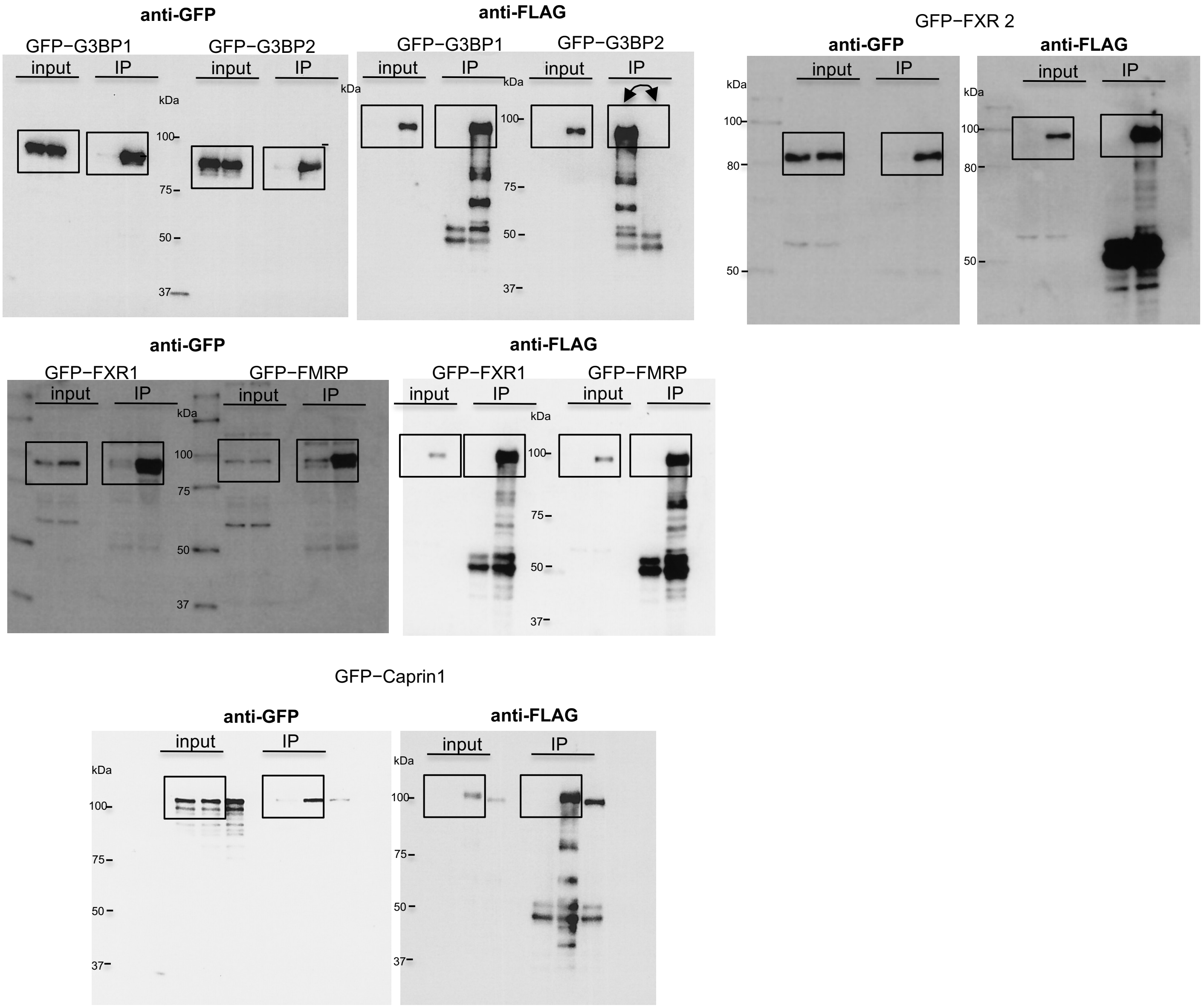

Uncropped blots for Figure 3c and supplementary Figure 3b

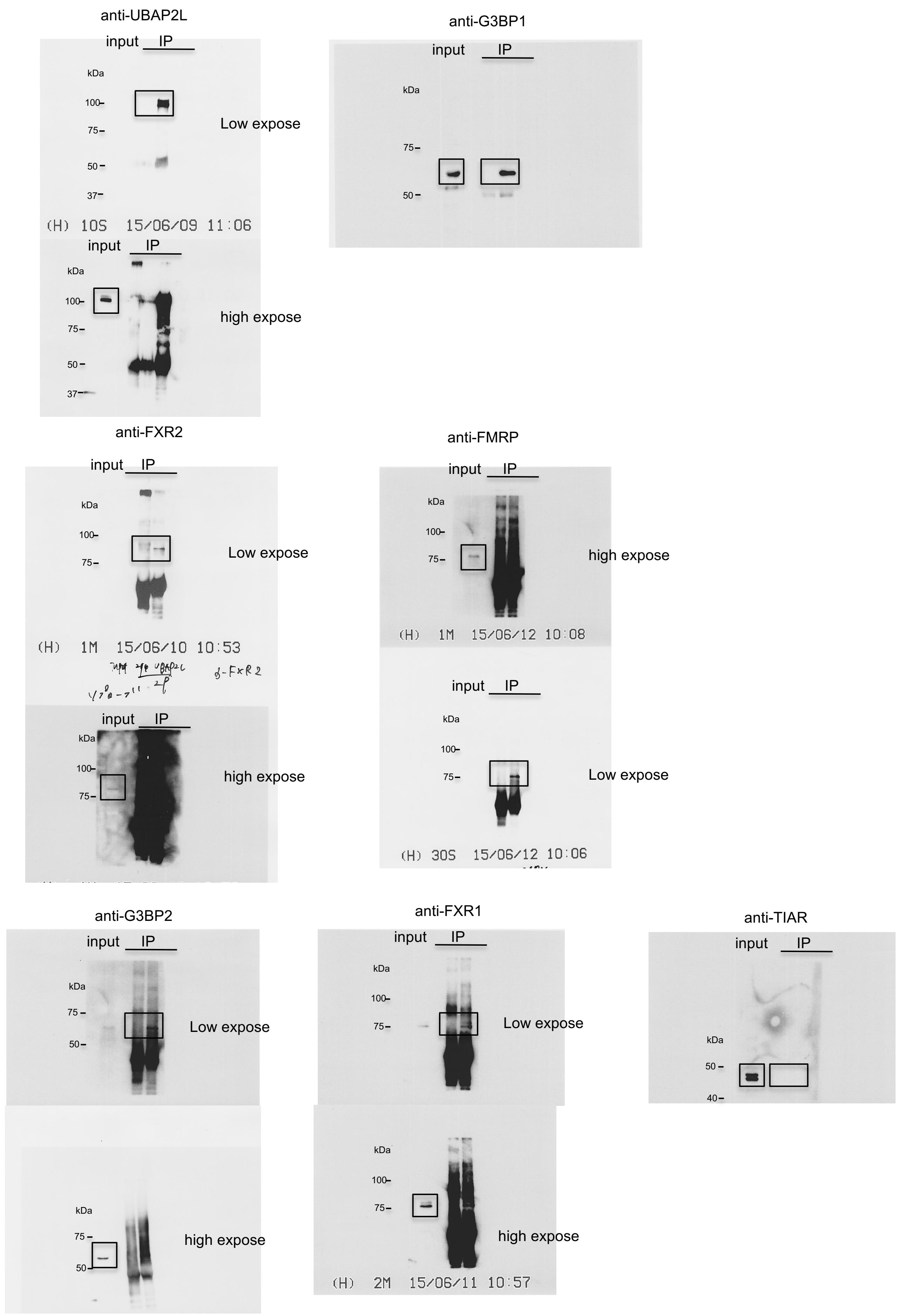

Uncropped blots for supplementary Figure 3c

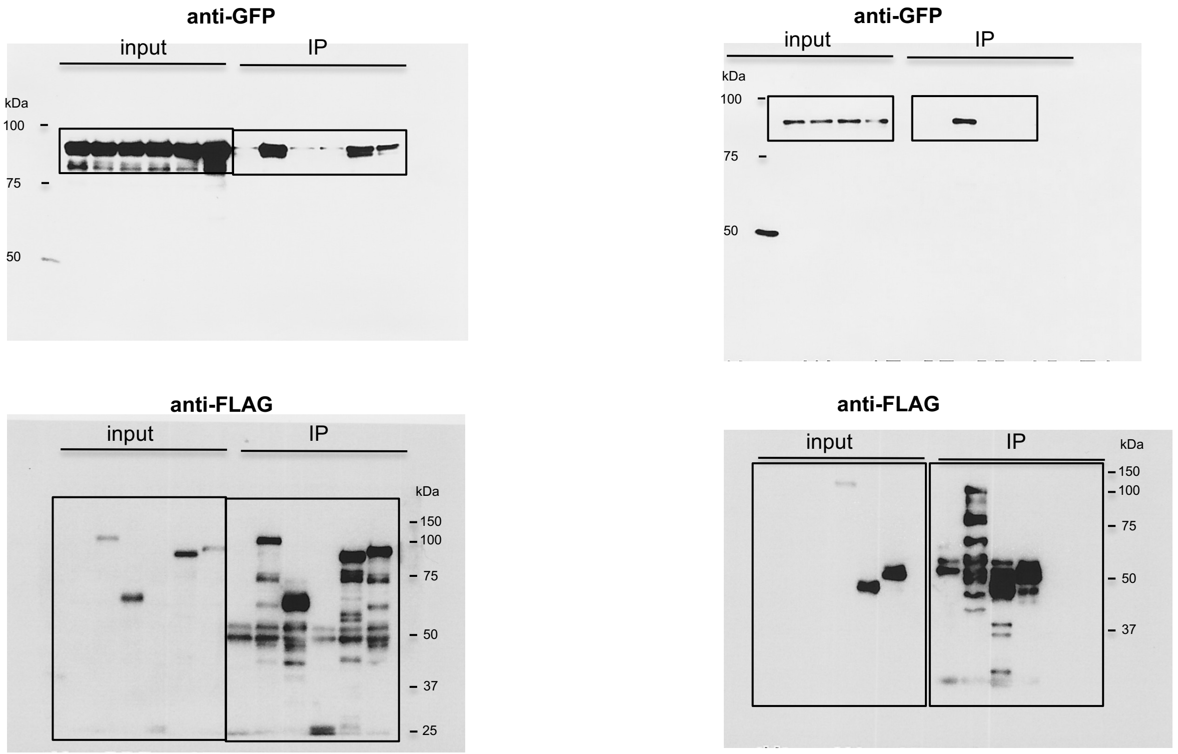

Uncropped blots for supplementary figure 3d

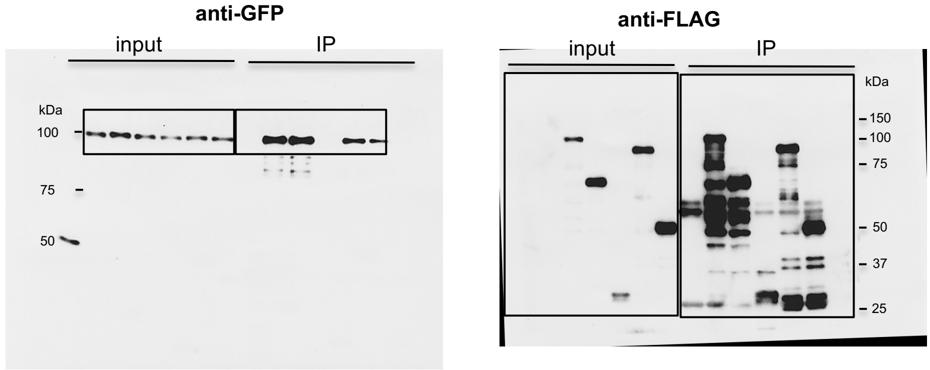

Uncropped blots for Figure 3f

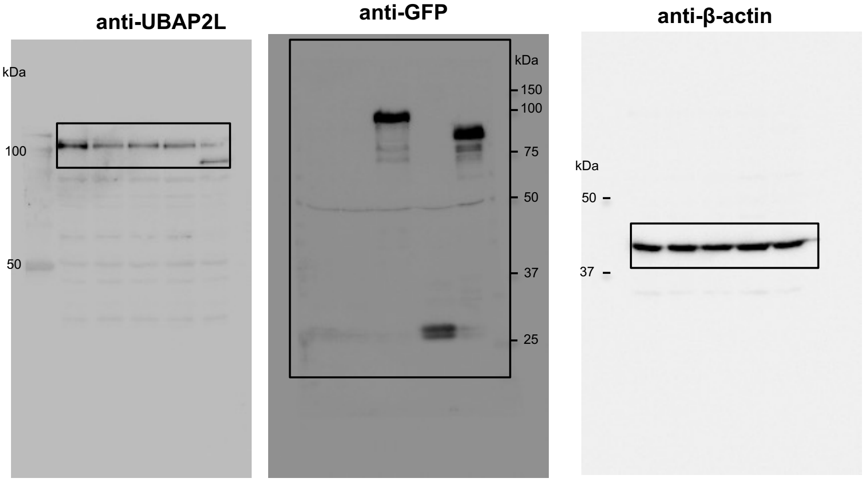

Uncropped blots for Figure 4a

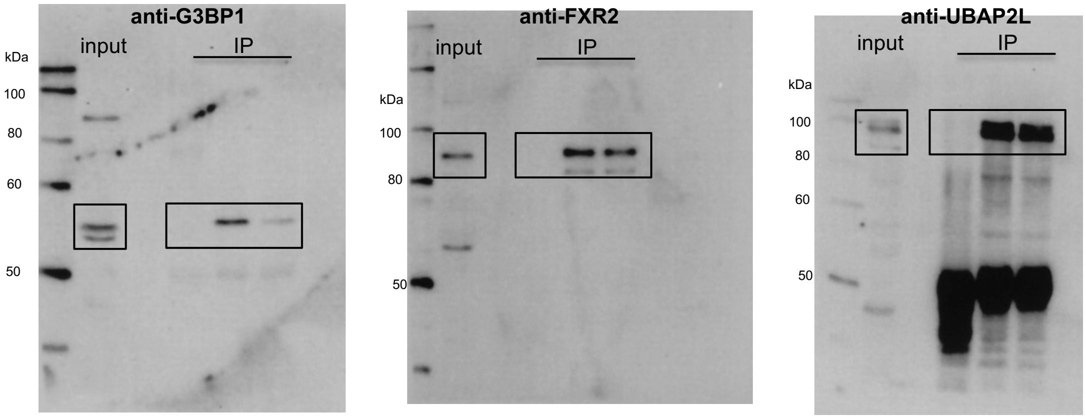

Uncropped blots for Figure 4b and supplementary Figure 4c

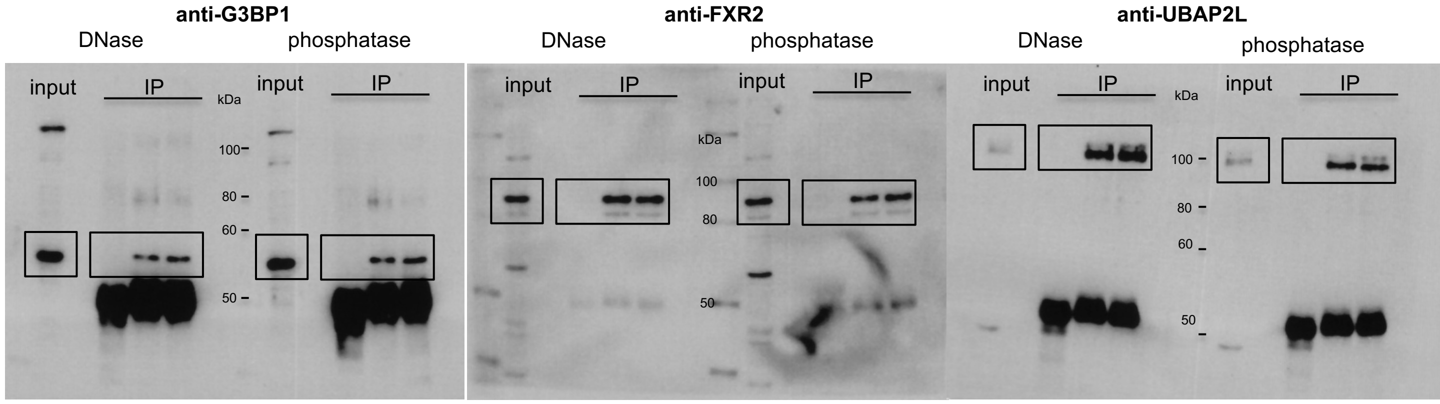

Uncropped blots for Figure 4c

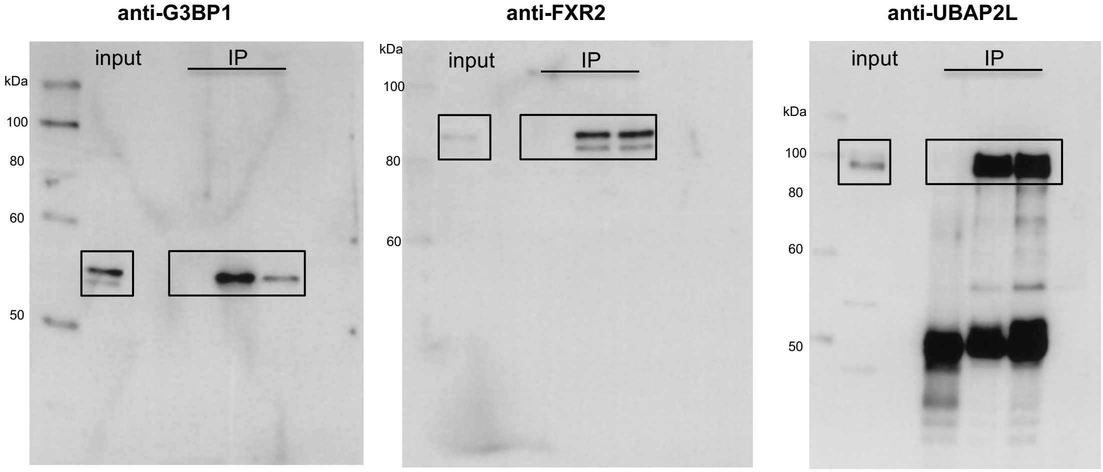

Uncropped blots for supplementary Figure 4a

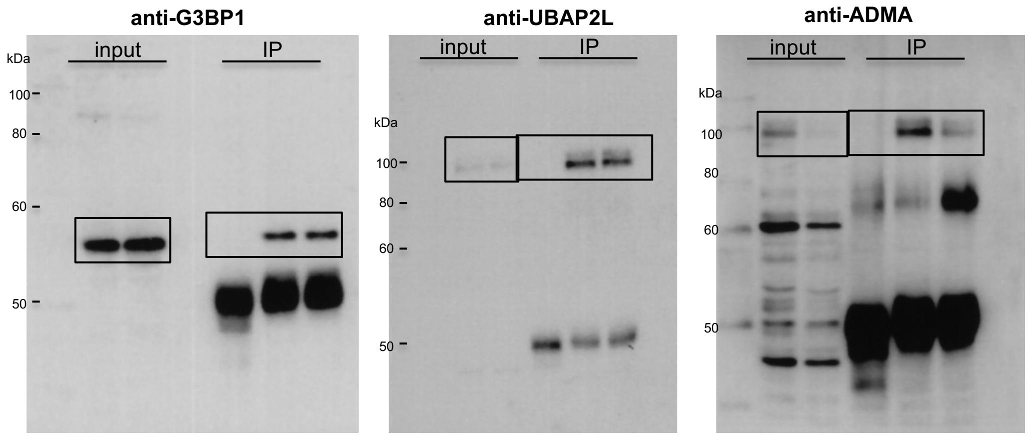

Uncropped blots for Figure 4f

anti-FLAG

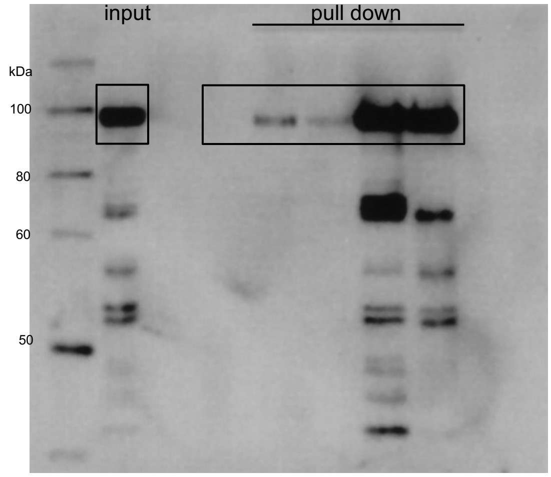

CBB

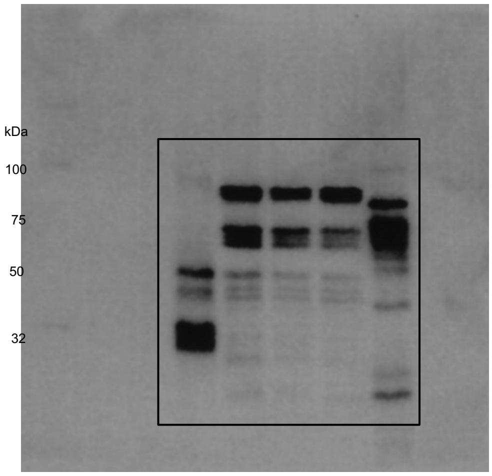

Uncropped blots for Figure 5d

anti-FLAG

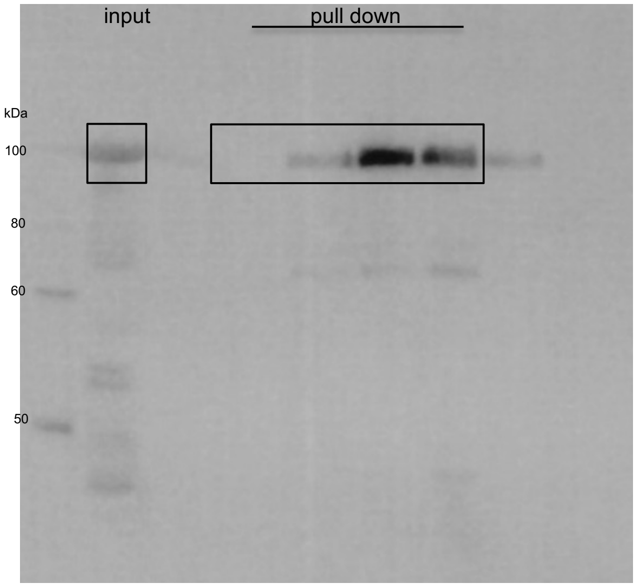

CBB

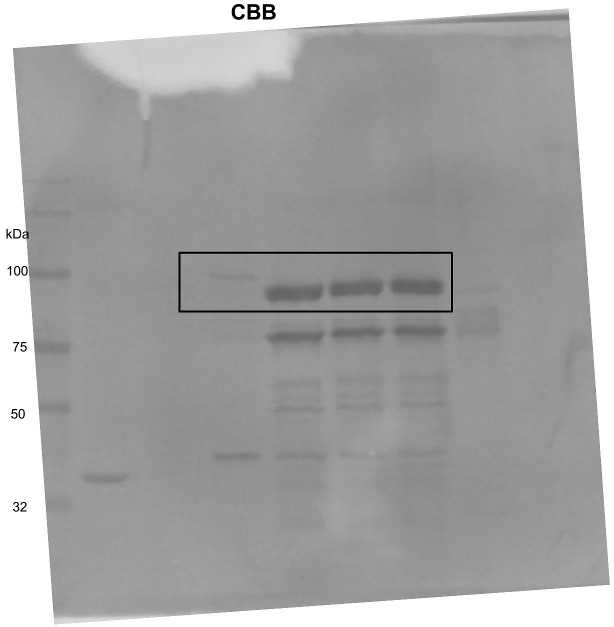

Uncropped blots for Figure 5e

anti-FLAG

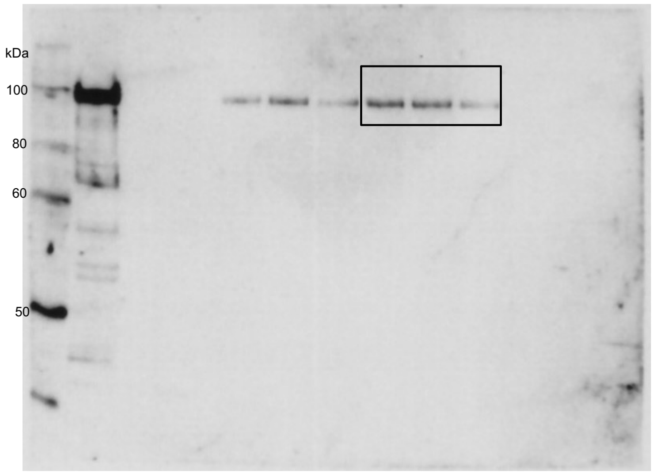

CBB

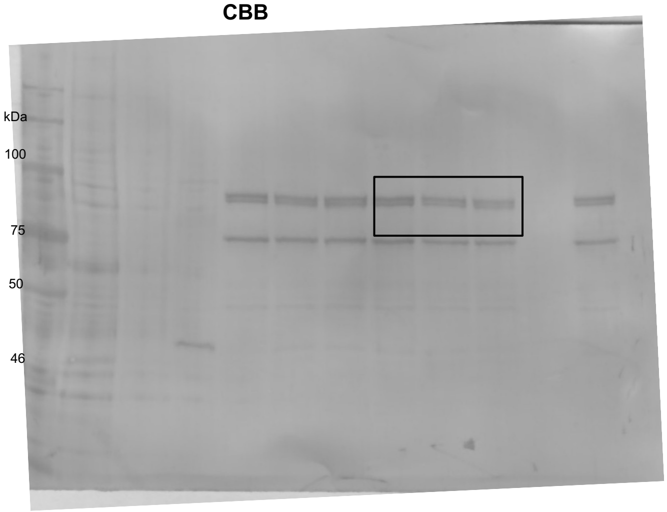

Uncropped blots for Figure 6e

anti-UBAP2L

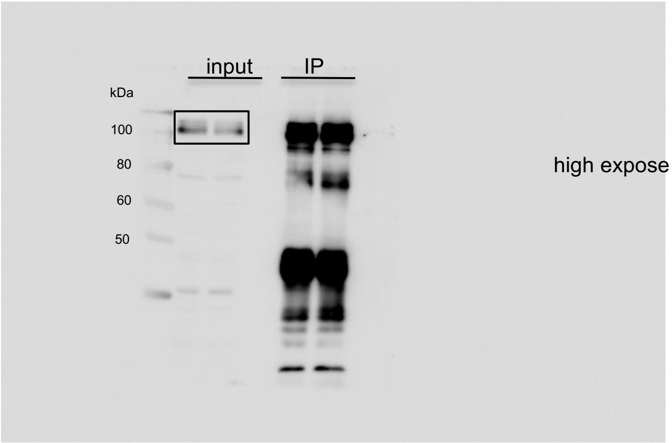

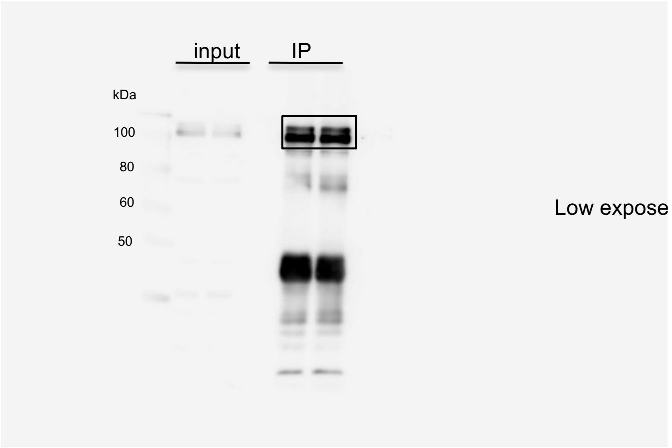

anti-G3BP1

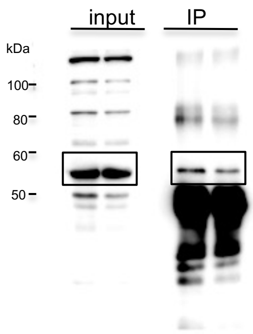

Supplement: Supplementary file 2 — Supplementary Information [file 42003_2023_4754_MOESM2_ESM.pdf]
