# Supplementary material for: The association of UBAP2L and G3BP1 mediated by small nucleolar RNA is essential for stress granule formation
Source: Commun Biol. 2023 Apr 14;6:415. doi: 10.1038/s42003-023-04754-w (PMC10104854; doi:10.1038/s42003-023-04754-w)
Supplement: Supplementary file 3 — Description of Additional Supplementary Files [file 42003_2023_4754_MOESM3_ESM.pdf]

Description of Additional Supplementary Files

File name: Supplementary Data 1

Description: The low RNA-seq data behind the graphs in the figure 5 a and b
